# Metabolite Profiling of Sorghum Seeds of Different Colors from Different Sweet Sorghum Cultivars Using a Widely Targeted Metabolomics Approach

**DOI:** 10.1155/2020/6247429

**Published:** 2020-03-04

**Authors:** Yaxing Zhou, Zhenguo Wang, Yan Li, Zhigang Li, Hui Liu, Wei Zhou

**Affiliations:** ^1^Agricultural College, Inner Mongolia University for Nationalities, Tongliao, 028000 Inner Mongolia, China; ^2^Tongliao Academy of Agricultural Science, Tongliao, 028000 Inner Mongolia, China; ^3^Independent Researcher, Chifeng, 024000 Inner Mongolia, China

## Abstract

Sweet sorghum (*Sorghum bicolor*) is one of the most important cereal crops in the world with colorful seeds. To study the diversity and cultivar-specificity of phytochemicals in sweet sorghum seeds, widely targeted metabolomics was used to analyze the metabolic profiles of the white, red, and purple seeds from three sweet sorghum cultivars Z6, Z27, and HC4. We identified 651 metabolites that were divided into 24 categories, including fatty acids, glycerolipids, flavonoids, benzoic acid derivatives, anthocyanins, and nucleotides and its derivatives. Among them, 217 metabolites were selected as significantly differential metabolites which could be related to the seed color by clustering analysis, principal component analysis (PCA), and orthogonal signal correction and partial least squares-discriminant analysis (OPLS-DA). A significant difference was shown between the red seed and purple seed samples, Z27 and HC4, in which 106 were downregulated and 111 were upregulated. The result indicated that 240 metabolites were significantly different, which could be related to the purple color with 58 metabolites downregulated and 182 metabolites upregulated. And 199 metabolites might be involved in the red phenotype with 54 downregulated and 135 upregulated. There were 45 metabolites that were common to all three cultivars, while cyanidin O-malonyl-malonyl hexoside, cyanidin O-acetylhexoside, and cyanidin 3-O-glucosyl-malonylglucoside were significantly upregulated red seeds, which could be the basis for the variety of seed colors. Generally, our findings provide a comprehensive comparison of the metabolites between the three phenotypes of *S. bicolor* and an interpretation of phenotypic differences from the point of metabolomics.

## 1. Introduction

Sweet sorghum (*Sorghum bicolor*) is one of the most important cereal crops in the world. It is remarkable for its resistance to stress and its extremely high photosynthetic efficiency; therefore, sweet sorghum could achieve high yields when it is widely grown in harsh environments such as arid or semiarid areas [1, 2]. Sweet sorghum is not only a crop but also a bioenergy source for producing ethanol. Compared with sugar cane, the cost of producing ethanol from sweet sorghum is reduced to 20-56%; therefore, it is used as the substitute for corn, cassava, and other crops. Besides, sorghum can also make wine and sugar. The sorghum original wine tastes mellow, accounting for more than 90% of the liquor wine market. Furthermore, sorghum is a raw material to make sugar as a substitute for sugar cane [3].

Metabolites, including primary metabolites and secondary metabolites, play a very important role in the growth and development of plants. Primary metabolites are necessary for life-sustaining activities and growth. Despite high levels of primary metabolites, there are only a few species. Unlike primary metabolites, secondary metabolites are more involved in response to plant disease, stress, and other forms of environmental stimulation. Among them, flavonoids, including flavones, flavonols, anthocyanins, flavanones, and chalcones, are a class of important plant secondary metabolites. Many colored fruits and flowers contain a large amount of flavonoids, which are conducive for protection against damage by dormancy, ultraviolet light, and phytopathogens, and are free from biotic and abiotic stresses [4]. Researchers have shown that due to their antioxidant activities, flavonoids are very helpful to some patients with inflammatory diseases, chronic diseases, and certain types of cancer [5]. Recently, a subgroup of flavonoids, anthocyanins, has been paid more and more attention. More than 600 anthocyanins have been identified from fruits or flowers [6–8]. Anthocyanin and its derivatives such as delphinidin, petunidin, malvidin, yanidin, and pelargonidin could make purple and dark colors in bright-red-colored fruit [9]. These flavonoids may be the material basis of different colors, and plants of different colors contain different types of anthocyanins or flavones. For example, the red color of strawberry is determined by anthocyanins, and the purple color of blueberry is determined by flavonoids. The red color of tomato is determined by carotenoids. The color of sweet sorghum seeds varies greatly [10–12]; however, few studies have systematically compared the metabolite differences between these varieties from the perspective of metabolomes. In this paper, targeted metabolome technology was used to compare three different sweet sorghum seed metabolites in an attempt to determine the characteristic metabolites of seed color formation. Our study is helpful for revealing the pigment accumulation of sweet sorghum seeds, as well as the formation and regulatory mechanisms of metabolites, which is of great significance for the study of sweet sorghum seed coat color.

## 2. Materials and Methods

### 2.1. Plant Materials

Three local sweet sorghum cultivars (namely Z6, Z27, and HC4) of Tongliao City of Inner Mongolia, China with excellent quality characteristics were chosen. The seeds of these three cultivars are white (Z6), red (Z27), and black (HC4), respectively. The experiment was conducted at the Experimental Base of the Agricultural College of Inner Mongolia University for Nationalities (N 42°15′-45°41′, E 119°15′-123°43′). The number of days with gales above level 8 can reach 20 to 30 days. The soil in the experimental field is gray meadow sand (pH 8.3) with 26 g/kg organic matter, 62 mg/kg alkali nitrogen, 38 mg/kg quick-acting phosphorus, and 184 mg/kg quick-acting potassium. The trial used a randomized block design with 3 replicates in planting 16 rows per plot, with a length of 5 m, a row spacing of 0.25 m, a plot area of 20 m^2^, and a spacing of 0.5 m. Seeding is carried out by means of aerial seeding. Compound fertilizer was applied at the time of sowing with 750 kg/hm^2^, and the others were consistent with the production management of farmland. It is bagged at the flowering stage, and a Mesh bag is an alternative at the end of the flowering period. After ripening, seeds from three types of sweet sorghum are harvested. All sweet sorghum cultivars were sown on April 28, 2018.

### 2.2. Sample Preparation and Metabolite Extraction

Seeds from each cultivar were collected from 5-6 plantlets as one sample. The collected seeds were washed three times with distilled water, naturally dried, and then stored at -80°C for further analysis.

The dried frozen seeds of one sample were mixed with zirconia beads then milled with a mixer (MM 400, Retsch) at 30 Hz for 1.5 min. For 100 mg of powder, 1 *μ*l precooled methanol (70% *v*/*v*) was added into a 1.5 ml microcentrifuge tube and vortexed and stored at 4°C overnight for extraction. Extraction mixtures were centrifuged for 10 min at 4°C, then the supernatants were filtrated (SCAA-104, 0.22 *μ*m pore size; ANPEL, Shanghai, China) and absorbed (CNWBOND Carbon-GCB SPE Cartridge, 250 mg, 3 ml; ANPEL, Shanghai, China) subsequently. Collected extraction samples were analyzed using an LC-ESI-MS/MS system (HPLC, Shim-pack UFLC Shimadzu CBM30A system; MS, Applied Biosystems QTRAP 6500).

### 2.3. Qualitative and Quantitative Principles of Widely Targeted Metabolomics and Data Analysis

Based on the self-built MetWare database (MWDB) and the metabolite information of public databases, the metabolites were subjected to qualitative analysis according to the secondary spectrum information. In the process of analysis, many things were deleted including the isotope signal; the repeated signals including K^+^ ions, Na^+^ ions, and NH4^+^ ions; and repetitive signals of fragment ions from large molecular weight substances. Metabolite quantification was carried out using multiple reaction monitoring (MRM) in triple quadrupole mass spectrometry. In the MRM mode, triple quadrupole mass spectrometry first screens the precursor ions (parent ions) of the target substance and excludes the ions corresponding to other molecular weight substances to initially eliminate the interference. The precursor ions pass through the collision chamber which induces dissociation and break to form a lot of fragment ions. Then triple quadrupole mass spectrometry is utilized to select a desired feature fragment ion to eliminate nontarget ion interference, which makes the quantification more accurate and repeatable. After obtaining metabolite mass spectrometry data from different samples, peak area integration is performed on the mass spectrum peaks of all the substances, so that the mass spectrometry peaks of the same metabolite in different samples are integrated for correction [13].

Characteristic ions of each substance are screened by the triple quadrupole mass spectrometer. The signal intensity (CPS) of the characteristic ions is obtained in the detector, and the mass spectrometry file of the sample is opened by MultiQuant software. The peak area of each chromatogram peak represents the relative content of the corresponding metabolites, and finally all of the chromatographic peak area integral data are stored. In order to compare the differences of the substance content of each metabolite in different samples among all detected metabolites, according to the information of metabolite retention time and peak type, we corrected the detected mass spectrum peaks of each metabolite in different samples to ensure the accuracy of qualitative and quantitative analyses. Supplementary [Supplementary-material supplementary-material-1] shows the integration correction results of randomly selected metabolites in different samples.

The quality control sample (QC) is prepared by mixing sample extracts and is used to analyze the repeatability of the sample under the same treatment method. During the analysis, a quality control sample is inserted into every 10 test analysis samples to monitor the repeatability of the analysis process.

By performing an overlapping display analysis of the total ion chromatogram (TIC) map of mass spectrometric detection of different QC samples, the repeatability of metabolite extraction and detection can be judged as well as technical duplication. The extraordinary stability of the instrument provides an important guarantee for data repeatability and reliability (Supplementary [Supplementary-material supplementary-material-1]).

Principle component analysis (PCA) and partial least squares-discriminant analysis (PLS-DA) were performed to analyze metabolite cultivar-specific accumulation based on Wang et al.'s description [14]. Principal component analysis of samples (including quality control samples) was conducted to provide an overall understanding of the metabolic differences between groups and the degree of variation between samples within the group. The principal component analysis (PCA) analysis were performed as the previous description [15]. For the PLS-DA model, the prediction parameters of the evaluation model are R2X, R2Y, and Q2, where R2X and R2Y, respectively, represent the interpretation rate of the X and Y matrix, and Q2 represents the prediction ability of the model. The closer these three indicators are to 1, the more stable and reliable the model is. When Q2 > 0.5, it is considered as a valid model. When Q2 > 0.9, it is an excellent model [16]. Log 2 transformation of metabolite relative response values was conducted, then the R package heat map was used to draw heat map plots. R (version 3.5.1) and SPSS software (version 19, IBM Corp., Armonk, NY, USA) were used to calculate the area under the receiver operating characteristic (AUC) values, sensitivity (SE), and specificity (SP) of the potential biomarkers to evaluate the differential performance of metabolites [17].

The Kyoto Encyclopaedia of Genes and Genomes (KEGG) pathway analysis was performed according to the previous study [18].

## 3. Results

### 3.1. Agronomic Traits of Three Sweet Sorghum Cultivars

**Figure 1:**
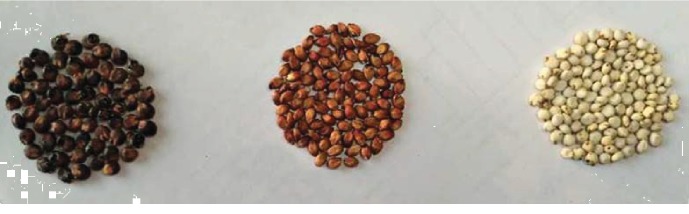
Seed color of the three sweet sorghum cultivars.

As shown in Table 1, the Z6 group had a longer growth period; the time from emergence to heading, the flowering period, and the time from emergence to flowering days were also longer compared with those of Z27 and HC4. There are different spike stalk protruding states, ear shapes, and seed colors in the three groups; in particular, the seed color varies greatly (Figure 1)—Z6 is white and Z27 and HC4 are red and black.

### 3.2. PCA and OPLS-DA Analysis

**Figure 2:**
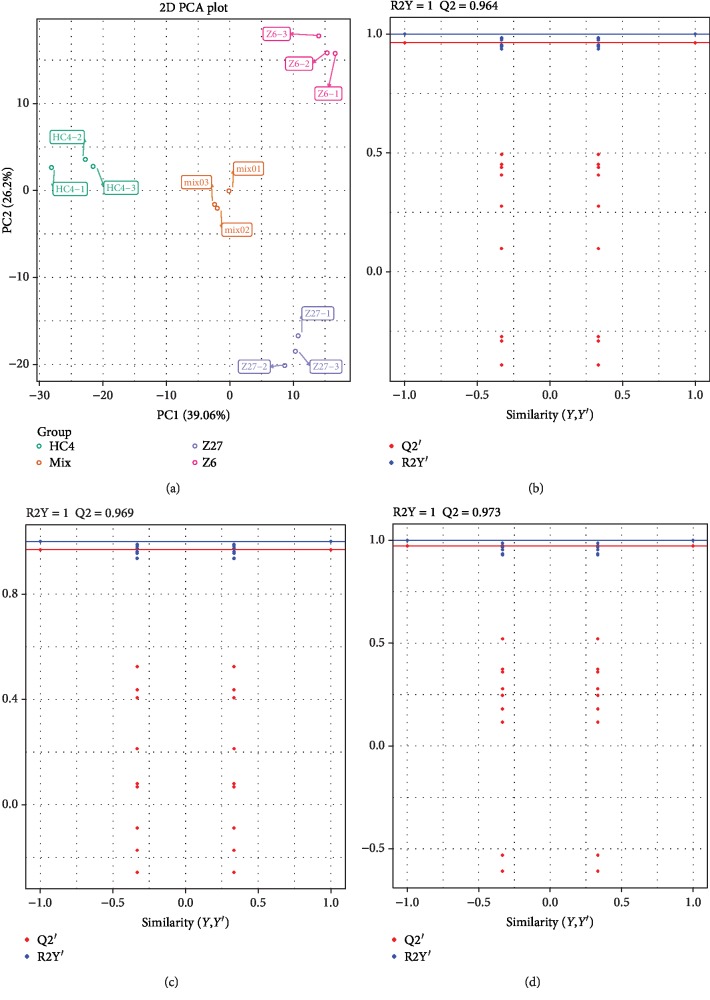
OPLS-DA and PCA. (a) PCA score plot. OPLS-DA model plots for the following comparison groups: (b) between Z6 and Z27, (c) between Z6 and HC4, and (d) between Z27 and HC4.

Principal component analyses of samples (including quality control samples) provide an initial understanding of the overall metabolic differences between groups and the degree of variability between samples within the group. The PCA analysis result showed that two principal components (PC1, PC2) separately account for 39.06% and 26.2% and three groups were distinctly separated, and the repeated samples were compactly gathered together; thus, these data indicate that the experiment was reproducible and reliable (Figure 2(a)). Orthogonal Partial Least Squares-Discriminant Analysis (OPLS-DA) [16] that maximizes the distinction in different groups was used to find differential metabolites. As shown in Figure 2, the value of Q2 between Z6 and Z27 was 0.964, that of Z6 and HC4 was 0.969, and that of Z27 and HC4 was 0.973 (Figure 2). These results showed that these models were reliable and could be conducted to screen for differential metabolites.

### 3.3. Metabolic Profiles and Flavonoid Expression

**Figure 3:**
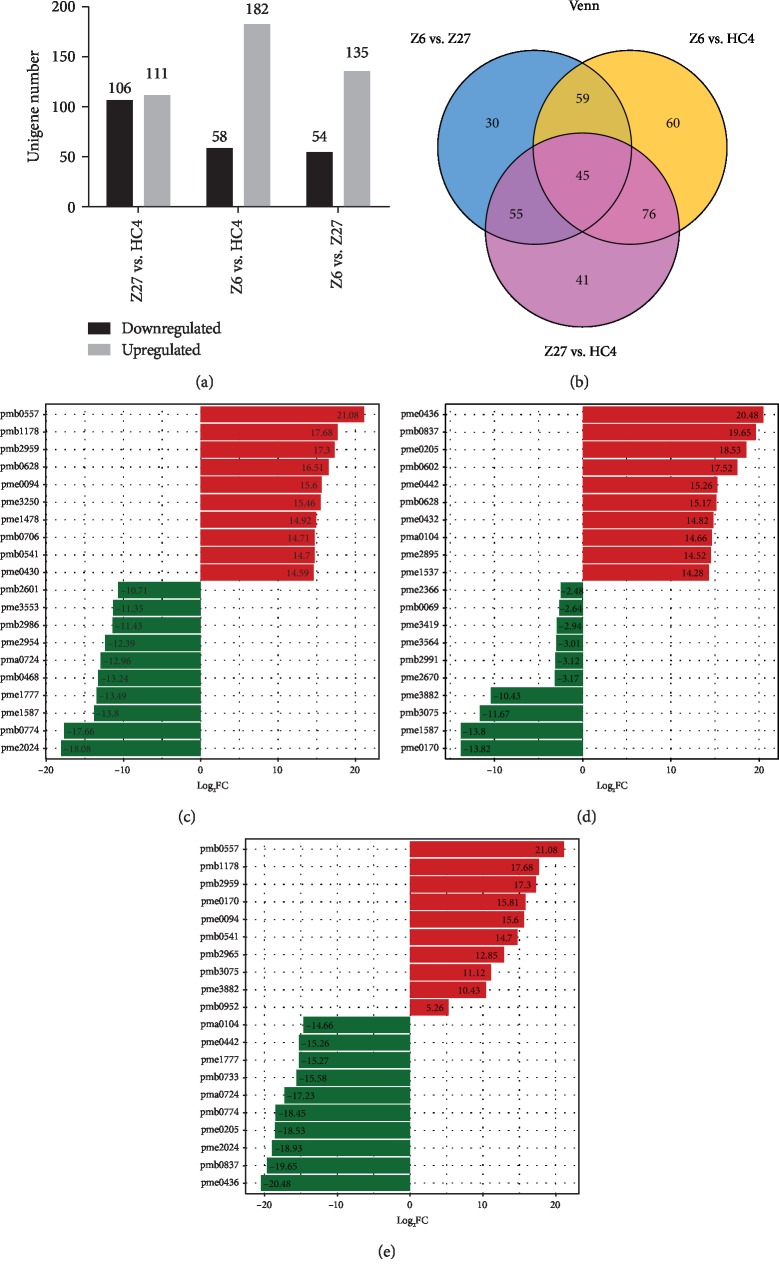
Numbers of significantly differentially accumulated metabolites, Venn diagram, and 10 most different metabolites in the comparison groups. (a) DAMs are shown in light color (upregulated) and black (downregulated) between Z27 and HC4, between Z6 and HC4, and between Z6 and Z27, respectively. (b) Venn diagram result between the three groups. (c–e) 10 most different metabolites in the comparison groups between Z6 and HC4, between Z6 and Z27, and between Z27 and HC4 in red color (upregulated) and blue (downregulated).

From the obtained multivariate analysis of the variable importance in project (VIP) of the OPLS-DA model, metabolites in the three varieties and differences between tissues can be initially screened. At the same time, the differential metabolites can be further screened by combining the *p* value or the fold change. A total of 366 differential metabolites were found, including 217 significantly different metabolites between Z27 and HC4 (106 downregulated, 111upregulated), 240 between Z6 and HC4 (58 downregulated, 182upregulated), and 199 between Z6 and Z27 (54 downregulated, 135 upregulated) (Figure 3(a)). Venn diagram analysis showed that 45 differential metabolites were the same for all three comparison groups (Figure 3(b)).

The difference fold change in metabolite quantitative information in each group was compared in combination with the grouping of specific samples. The most different metabolites in comparison groups are shown in Figures 3(c)–3(e). The results of Z6 vs. HC4 indicated that cyanidin O-malonyl-malonylhexoside, O-feruloyl 2-hydroxylcoumarin, cyanidin O-acetylhexoside, eriodictiol C-hexosyl-O-hexoside, cyanidin 3-O-glucoside (kuromanin), biochanin A, myricetin, quercetin 5-O-malonylhexosyl-hexoside, cyanidin 3-O-glucosyl-malonylglucoside, and procyanidin A1 were the most expressed metabolites, which belong to anthocyanins, coumarins, flavone C-glycosides, isoflavones, and proanthocyanidins. The most expressed metabolites in HC4 were tryptamine derivatives, isoflavones, anthocyanins, amino acid derivatives, flavone C-glycosides, flavonols, flavones, coumarins, and benzoic acid derivatives. The results of additional comparison groups are listed in Supplementary Tables [Supplementary-material supplementary-material-1], [Supplementary-material supplementary-material-1] and [Supplementary-material supplementary-material-1].

In addition, we obtained a total of 651 metabolites, of which 165 were flavonoids, accounting for a quarter of the total metabolites. The differential metabolites between Z6 and HC4 are 67 kinds of flavonoids. Most of the upregulated expressions in HC4 are similar in Z6 and Z27. The results showed that flavonoids were higher in dark seeds compared to light seeds.

**Figure 4:**
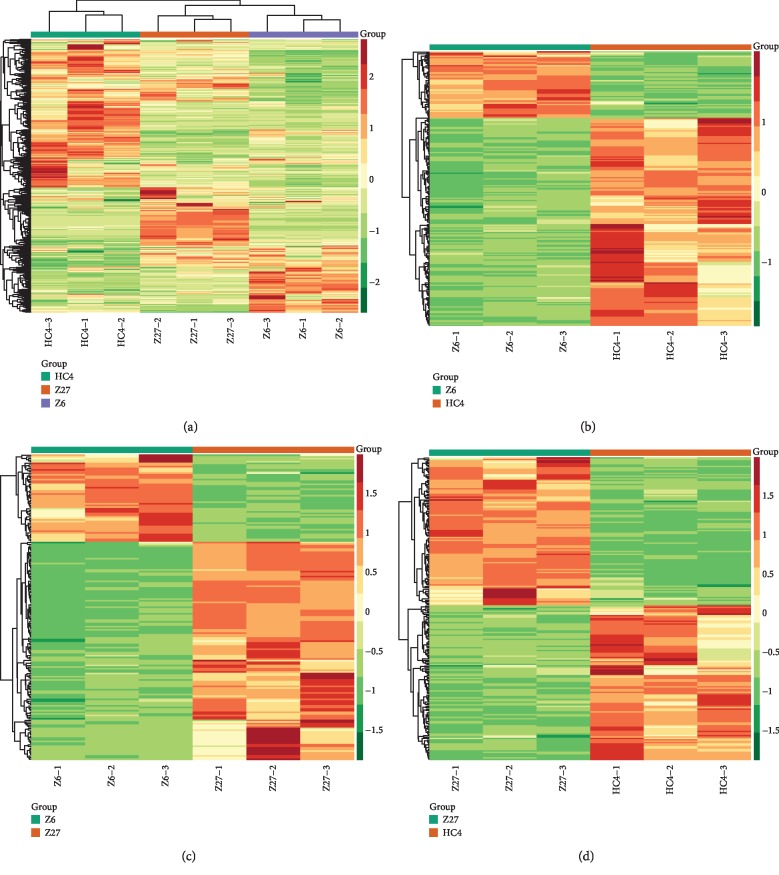
Cluster heat map for all metabolites and the differential metabolite heat maps for the comparison groups. (a) The heat map of all metabolites. The differential metabolite heat maps of the following comparison groups: (b) between Z6 and HC4, (c) between Z6 and Z27, and (d) between Z27 and HC4.

In order to explore the expression trend of metabolites in different groups of metabolites, we performed heat map analysis based on the relative relationship of the metabolite accumulation patterns of the three groups of samples. The results showed that three major clusters of metabolites were formed in the three groups of samples (Figure 4(a)). Metabolites of cluster 1 were mainly in HC4, while the metabolites within clusters 3 and 4 had higher levels in Z27 and Z6, respectively. We then compared the trends of differential metabolites in any two groups, and the results showed that the differential metabolites were significantly different between the comparison groups (Figures 4(b)–4(d)).

**Figure 5:**
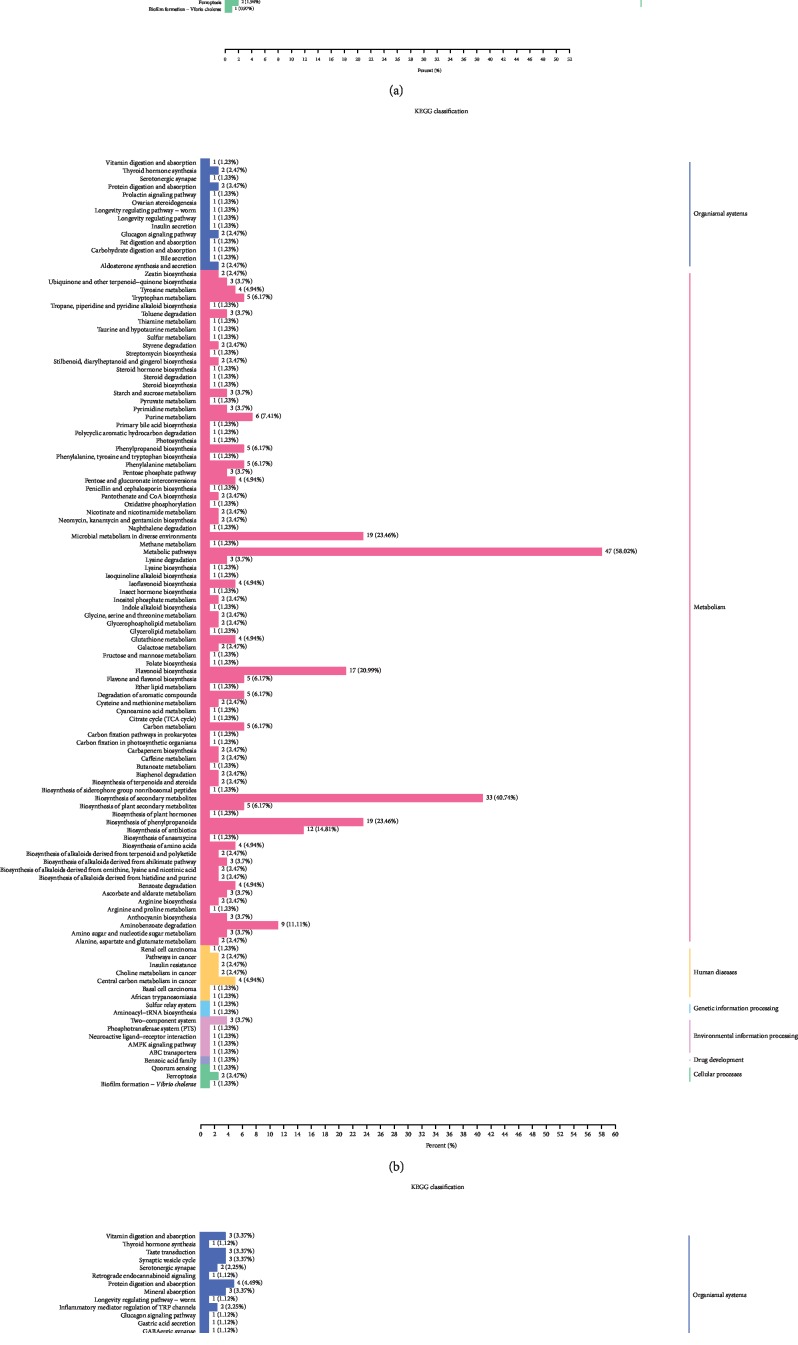
KEGG classification results. The differential metabolites of KEGG classification of the following comparison groups: (a) between Z27 and HC4, (b) between Z6 and HC4, and (c) between Z6 and Z27.

KEGG pathway analysis was carried out to integrate genes, expressions, and metabolites as a whole for research. The results of the annotation of the significant metabolite KEGG were ranked according to the type of pathway in KEGG. As shown in Figure 5, many differential metabolites were found in metabolic pathways and in secondary metabolite biosynthesis.

## 4. Discussion

Sweet sorghum is cultivated throughout China, but it is mostly in the south of the Yellow River Basin. It is the lowest cost bioenergy source and can be utilized to replace corn, cassava, and other crops. Meanwhile, it is well suited for use as a raw material for papermaking. It is also a new type of renewable and efficient energy crop [19, 20]. In this study, we compared three different sweet sorghum varieties grown at the same time and place. Many of their agronomic traits, including time of flowering, growth period, seeding leaf color, and seed color were different (Figure 1). Especially, the color of the seed of different varieties changes significantly. Some studies found that the seed coat contains different anthocyanins and flavonoids, which have a strong antioxidant capacity, so they can protect seeds and cause color change. Our study of metabolite profiles of seeds of different sweet sorghum varieties can provide some clues for explaining the mechanism of color change of sweet sorghum seeds.

Plant metabolomics, as a new direction in the postgenomic era [21], plays an important role in revealing the mechanism of metabolite changes in different plant tissues. The production of metabolites is a response to genetic and environmental changes [22]. Therefore, metabolomics analysis is a very useful tool to clarify the relationship between biological processes and phenotypes as well as intuitive changes in metabolic levels [23]. Many studies have been reported to explore the mechanism of the relationship between biological processes and phenotypes [24, 25]. In the current study, 651 metabolites were identified and further divided into 24 categories, including lipids-fatty acids, lipids-glycerolipids, flavonoids, benzoic acid derivatives, and anthocyanins (Supplementary [Supplementary-material supplementary-material-1]). PCA and heat map of the three groups showed a distinctive separation between the three cultivars. The results showed that the metabolites contained in the three groups were very different. In order to further explore the mechanism of seed color differences between different cultivars, the differentially expressed metabolites were analyzed between any two groups. There are 217 significantly different metabolites between Z27 and HC4, 240 between Z6 and HC4, and 199 between Z6 and Z27. KEGG results showed that there were more differential metabolites in metabolic pathways and biosynthesis of secondary metabolites (Figure 5). Secondary metabolites are mainly related to changes in the color of flowers and seeds and have a great effect on resistance to external aggression [26–28], which is consistent with many previous reports [29, 30]. Anthocyanins and flavonoids determine the color and taste of fruits. They have antioxidant and nutritional health functions and are reported to reduce the incidence and mortality of cardiovascular disease [31, 32]. Of the 651 metabolites we identified, 165 were flavonoids, accounting for a quarter of all metabolites. 67 flavonoids were differential expressions between Z6 vs. HC4, and the vast majority were upexpressions in HC4, which has a similar expression pattern with that of Z6 vs. Z27. The results suggested that flavonoids were higher in dark seeds compared with light-colored seeds. However, in Z27 vs. HC4, the type and amount of flavonoids are different, and eventually different seed colors are formed. Anthocyanins are water-soluble plant pigments, and they are the main causes of different colors (such as white, red, black, and blue) in vegetables, seeds, fruits, and flowers. Anthocyanins can also help plants prevent damage caused by harsh environments such as cold and drought and avoid damage caused by fungi, bacteria, and viruses, as well as attract pollinators and help in seed dispersal [26–28]. Cyanidin-3-O-rhamnoglucoside (cyanidin-3-O-rutinoside) was reported to be the main anthocyanin in the peel of “Black Mission” and “Brown Turkey” figs [33, 34]. Acyl-modified anthocyanins are common in *Arabidopsis* [35], and cyanidin 3-O-(malonyl)-glucoside was found in the cool-cultivated red lettuce responding to temperature [36]. Comparison of two cranberry cultivars showed that berries with high contents of pigments also had higher contents of colorless flavonol [37]. In our study, compared with Z27 (red) and Z6 (white), the content of anthocyanins (pmb0557 cyanidin O-malonyl-malonylhexoside, pmb2959 cyanidin O-acetylhexoside, and pmb0541 cyanidin 3-O-glucosyl-malonylglucoside) in HC4 (black) is the largest, with the largest difference multiple in the each of two comparison groups (Figures 3(c) and 3(e)). pme0436 (procyanidin B3) and pmb0837 (procyanidin A3) had the largest difference in expression between Z6 (white) and Z27 (red), with high expression in Z27 (Figures 3(c) and 3(e)). We speculate that these anthocyanin differences are the main cause of seed color differences.

## 5. Conclusion

This study focused on the metabolic diversity of three sweet sorghum cultivars with white, red, and black seeds to elucidate the factors responsible for the differences in seed color. PCA and heat-map analyses of the three groups showed a distinguished separation between the three cultivars. It shows that metabolites of the three groups are significantly different. Differentially expressed metabolites were analyzed between any two groups and the most different metabolites in comparison groups mainly are anthocyanins and flavonoids. In particular, we found that the largest difference in metabolites between any two groups is anthocyanins. These findings have improved our understanding of the metabolic mechanisms accounting for differences in seed colors in different sweet sorghum cultivars.

## Figures and Tables

**Table 1:** Agronomic trait statistics of three sweet sorghum cultivars.

Name	Bud sheath color	Seeding leaf color	Tiller	Plant height (cm)	Stem diameter (cm)	Main spike length (cm)	Main shank length (cm)
Z6	Green	Green	No	240	1.4	18	30
Z27	Purple	Purple	No	289	1.2	28	45
HC4	Green	Green	No	298	1.4	25	55

Name	Spike out	Main pulse color	Spike shape	Shell color	Peridium rate	Seed color	Seed shape
Z6	Short	White	Cattle heart	Black	1/2	White	Circle
Z27	Long	White	Clavate	Black	1/2	Red	Oval
HC4	Medium	White	Proculiform	Black	1/2	Black	Oval

Name	Grain weight (g)	Dry grain weight (g)	Cutting rate (%)	Shelling rate (%)	Grain uniformity	Stem and leaf senescence	Sowing time
Z6	36	21	25	4%	Tidiness	No	5.8
Z27	25	24	30	2%	Tidiness	No	5.8
HC4	45	22	50	5%	Tidiness	No	5.8

Name	Sprout	Heading	Sprout-heading days	Flowering	Sprout-flowering days	Mature	Full growth period (days)
Z6	5.22	7.26	65	7.28	67	8.28	98
Z27	5.22	7.17	56	7.19	58	8.19	89
HC4	5.22	7.17	56	7.20	59	8.21	91

## Data Availability

The data used to support the findings of this study are included within the supplementary information files.

## Abbreviations

PCA: Principal component analysis
OPLS-DA: Orthogonal signal correction and partial least squares-discriminant analysis
MRM: Multiple reaction monitoring
PLS-DA: Partial least squares-discriminant analysis
SE: Sensitivity
SP: Specificity
KEGG: Kyoto Encyclopaedia of Genes and Genomes
LIT: Linear ion trap
QQQ: Triple quadrupole
CAD: Collision gas.

## Supplementary Materials

Supplementary 1 Figure S1: integral correction diagram for quantitative analysis of metabolites. The abscissa is the retention time (min) of metabolite detection. The ordinate is the ion flow strength (CPS) detected by a metabolite ion. The peak area represents the relative content of the substance in the sample.

Supplementary 2 Figure S2: multiple peaks of MRM metabolite detection. The abscissa is the retention time (Rt) of metabolite detection, and the ordinate is the ion flow strength (strength unit is count per second (CPS)) of ion detection.

Supplementary 3 Table S1: the metabolite and its corresponding content between Z6 vs. HC4.

Supplementary 4 Table S2: the metabolite and its corresponding content between Z6 vs. Z27.

Supplementary 5 Table S3: the metabolite and its corresponding content between Z27 vs. HC4.

Supplementary 6 Table S4: the metabolite and its corresponding content in Z6, HC4, and Z27.
