# Supplementary figures and images for: Metabolite Profiling of Sorghum Seeds of Different Colors from Different Sweet Sorghum Cultivars Using a Widely Targeted Metabolomics Approach
Source: Int J Genomics. 2020 Mar 4;2020:6247429. doi: 10.1155/2020/6247429 (PMC7073482; doi:10.1155/2020/6247429)

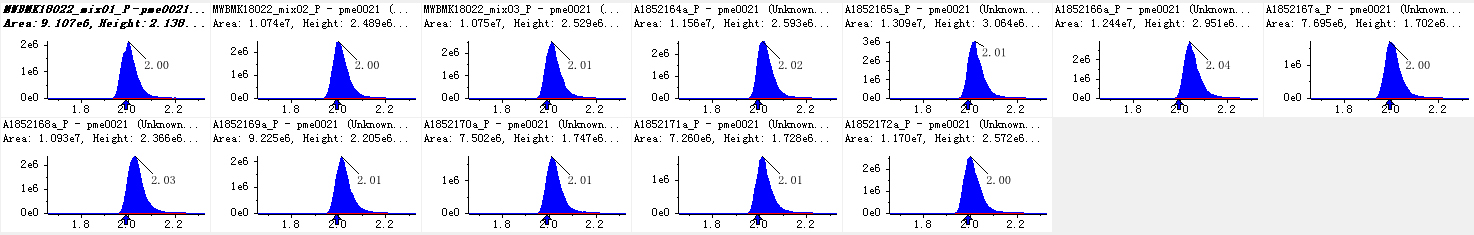

Supplement: Supplementary 1 — Figure S1: integral correction diagram for quantitative analysis of metabolites. The abscissa is the retention time (min) of metabolite detection. The ordinate is the ion flow strength (CPS) detected by a metabolite ion. The peak area represents the relative content of the substance in the sample. [file 6247429.f1.jpg]

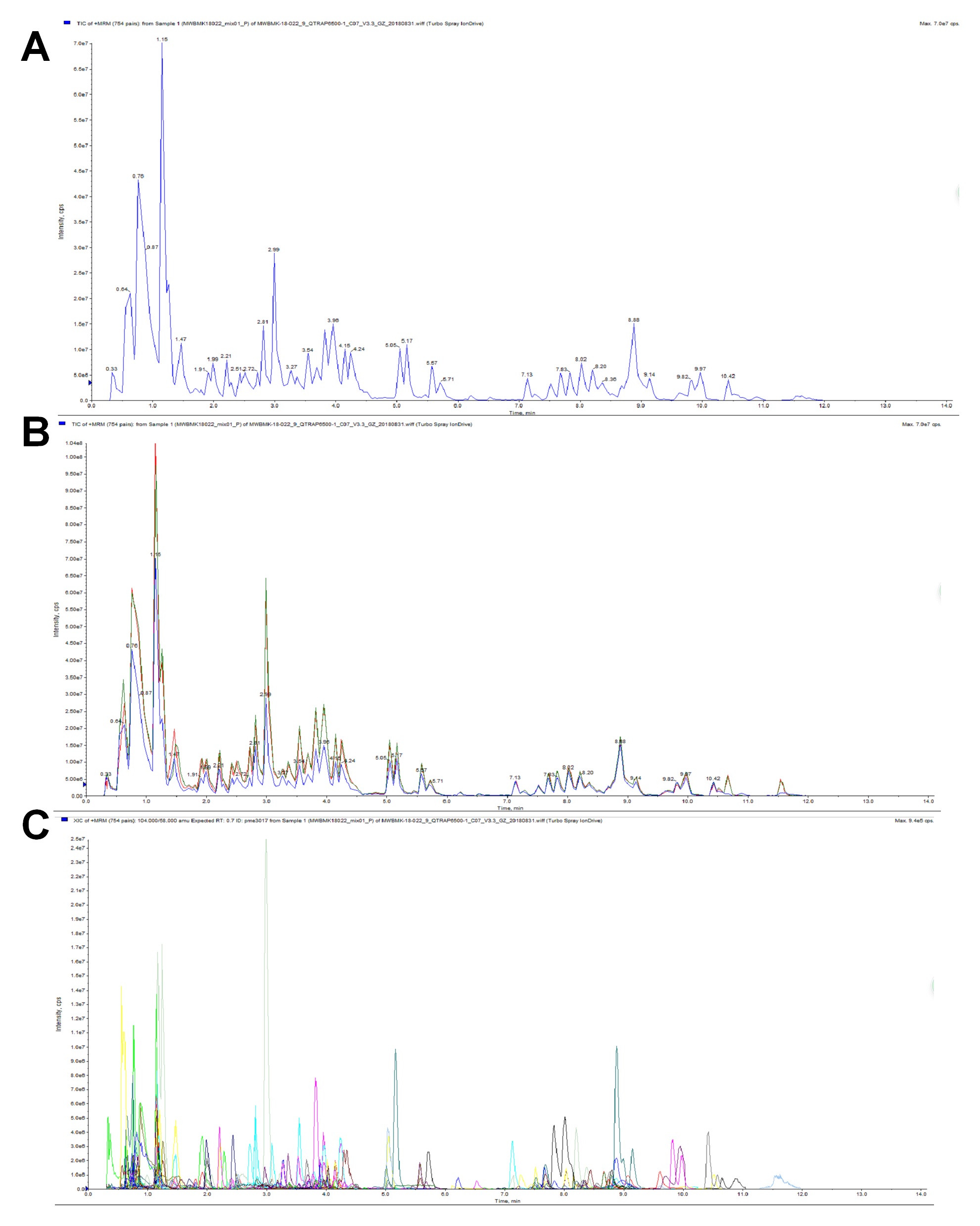

Supplement: Supplementary 2 — Figure S2: multiple peaks of MRM metabolite detection. The abscissa is the retention time (Rt) of metabolite detection, and the ordinate is the ion flow strength (strength unit is count per second (CPS)) of ion detection. [file 6247429.f2.jpg]
